# Whole Transcriptome RNA-Seq Reveals Drivers of Pathological Dysfunction in a Transgenic Model of Alzheimer’s Disease

**DOI:** 10.1007/s12035-025-04878-6

**Published:** 2025-04-05

**Authors:** Nikita Potemkin, Sophie M. F. Cawood, Diane Guévremont, Bruce Mockett, Jackson Treece, Jo-Ann L. Stanton, Joanna M. Williams

**Affiliations:** 1 Department of Anatomy, School of Biomedical Sciences, University of Otago, P.O. Box 56, Dunedin, New Zealand; 2 Brain Health Research Centre, Brain Research New Zealand–Rangahau Roro Aotearoa, University of Otago, Dunedin, New Zealand; 3 Department of Psychology, University of Otago, P.O. Box 56, Dunedin, New Zealand

**Keywords:** Alzheimer’s disease, RNA-Seq, Noncoding-RNA, APP/PS1, Bioinformatics

## Abstract

Alzheimer’s disease (AD) affects more than 55 million people worldwide, yet current theories cannot fully explain its aetiology. Accordingly, gene expression profiling has been used to provide a holistic view of the biology underpinning AD. Focusing primarily on protein-coding genes, such approaches have highlighted a critical involvement of microglia-related inflammatory processes. Simultaneous investigation of transcriptional regulators and noncoding RNA (ncRNA) can offer further insight into AD biology and inform the development of disease-modifying therapies. We previously described a method for whole transcriptome sampling to simultaneously investigate protein-coding genes and ncRNA. Here, we use this technique to explore transcriptional changes in a murine model of AD (15-month-old APP/PS1 mice). We confirmed the extensive involvement of microglia-associated genes and gene networks, consistent with literature. We also report a wealth of differentially-expressed non-coding RNA — including microRNA, long non-coding RNA, small nuclear and small nucleolar RNA, and pseudogenes — many of which have been overlooked previously. Transcription factor analysis determined that six transcription factors likely regulate gene expression changes in this model (*Irf8*, *Junb*, *c-Fos*, *Lmo2*, *Runx1*, and *Nfe2l2*). We then utilised validated miRNA-target interactions, finding 60 interactions between 15 miRNA and 42 mRNA (messenger RNA) with largely consistent directionality. Furthermore, we found that eight transcription factors (*Clock*, *Lmo2*, *Runx1*, *Nfe2l2*, *Egr2*, *c-Fos*, *Junb*, and *Nr4a1*) are likely responsible for the regulation of miRNA expression. Taken together, these data indicate a complex interplay of coding and non-coding RNA, driven by a small number of specific transcription factors, contributing to transcriptional changes in 15-month-old APP/PS1 mice.

**Supplementary Information:**

The online version contains supplementary material available at 10.1007/s12035-025-04878-6.

## Introduction

Alzheimer’s disease (AD) is a progressive degenerative disorder affecting an estimated 55 million people worldwide [1] and results in severe cognitive and memory deficits. Its characteristic pathology includes widespread cell death [2], extracellular aggregates of Amyloid-β peptide [3], and intracellular aggregates of hyperphosphorylated tau protein [4]. While significant strides have been made in understanding the aetiology and pathogenesis of AD, current theories cannot yet fully explain how or why the sporadic form of AD develops.

Important insights may therefore be gained by moving from a direct focus on amyloid-β and tau to a holistic view of the disease based on whole transcriptome sequencing and understanding the relationships between different RNA biotypes. Indeed, there is a growing recognition of the importance of non-coding RNA (ncRNA), which outnumber protein-coding genes in both number and expression, in both health and disease [5–7]. Already, many microRNA (miRNA), long non-coding RNA (lncRNA), and other RNA biotypes have been found to contribute to disease processes in AD. For example, several miRNAs influence the expression and downstream metabolism of the amyloid precursor protein (APP) [8–11], the expression and processing of tau protein [12–14], and processes of neuroinflammation [15–17]. Indeed, miRNA signatures are mooted to be useful diagnostic biomarkers of disease progression [18, 19]. Further, long non-coding RNA (lncRNA) has also been found to be differentially expressed in people with AD and in animal models [20]. These include β-secretase modifying lncRNA BACE1-AS [21], inflammation-associated SNHG1 and NDM29 [22, 23] and the promiscuous lncRNA NEAT1, which acts as a miRNA sponge [24] and an indirect regulator of gene expression through controlling nuclear retention [25] or DNA methylation [26]. Very little is currently known about other forms of ncRNA in AD as they are rarely studied; however, some small nucleolar RNA (snoRNA), small nuclear RNA (snRNA), and PIWI-interacting RNA (piRNA) play roles in processes that are disrupted in AD [27–29].

Key to understanding the role of ncRNA is understanding the relationships between RNA biotypes. However, to date, our knowledge of how the protein-coding and non-coding transcriptome interact is limited and derived from comparison of studies of protein-coding or non-coding transcriptomes in isolation. Accordingly, to examine the role of ncRNA and mRNA in AD, we developed a modified RNA-Seq protocol for identifying and quantifying both protein-coding and non-coding RNA from the same sample simultaneously. This method avoids biases introduced by the separate isolation of long and short RNA and the construction and sequencing of separate libraries for different RNA biotypes. Using this method, in the current study, we examined the entire transcriptome of the APP_swe_/PSEN1dE9 mouse model of AD, which includes a humanised transgene of APP bearing the Swedish mutation and a deletion of exon 9 on the human presenilin-1 gene. This model is widely used to imitate the early stages of the disease, including synaptic dysfunction, synaptic and neuronal loss, as well as plaque formation [30]. This allowed us to establish whole transcriptome profiles and examine expression profiles of both the miRNA and transcription factors and their targets in the same sample at the same time. This permitted simultaneous identification of functional relationships between RNA and the regulatory factors driving gene expression changes contributing to the pathological phenotype of the APP_swe_/PSEN1dE9 model mice.

## Methods

### Animal Studies

All animal use was compliant with the New Zealand Animal Welfare Act 1991 and performed under guidelines and approval of the University of Otago Animal Ethics Committee (approval number DET09/15). The reporting in this manuscript follows the recommendations in the Animal Research: Reporting on In Vivo Experiments (ARRIVE) guidelines [31]. In this study a double transgenic model of Alzheimer’s disease was used (APPswe/PS1dE9, B6C3 background, hereafter referred to as APP/PS1). These mice were originally sourced from The Jackson Laboratory (https://www.jax.org/strain/004462) and maintained as a colony at the University of Otago breeding facility. Animals underwent no additional procedures prior to their stated use. All mice were genotyped for the presence of human exon-9-deleted variant PSEN1, which co-segregates with the APPswe gene, as previously described [32]. For RNA-Seq, male transgenic (tg) and wild-type (wt) littermates at 15 months old (*n* = 4 per group) were anaesthetised with sodium pentobarbitol and the brains were removed into ice-cold artificial cerebrospinal fluid solution (aCSF; in mM: 124 NaCl, 3.2 KCl, 1.25 NaH_2_PO_4_, 26 NaHCO_3_, 2.5 CaCl_2_, 1.3 MgCl_2_, 10 d-glucose). The left hippocampus was dissected and snap-frozen on dry ice. All samples were stored at –80 °C until used.

### RNA Sequencing

Sequencing was performed as previously described [33]. Briefly, total RNA was extracted from the hippocampi of male 15-month-old APP/PS1 tg mice and wt littermates (*n* = 4 pairs) using the mirVana™ PARIS™ RNA isolation kit (Invitrogen; Cat #AM1556). Libraries were prepared using slight alterations to the Ion Total RNA-Seq kit v2 (Life Technologies; Cat #4479789), with ribosomal RNA removal by QIASeq FastSelect (Qiagen; Cat #334386). Pooled equimolar libraries underwent emulsion polymerase chain reaction (PCR) with the Ion OneTouch™ 2 system using the Ion PI™ Hi-Q OT2 200 kit (Invitrogen; Cat #A26434). Two pools of mixed barcoded libraries were sequenced on two Ion PI™ v3 chips (Invotrogen; Cat #A26772), with two mixed pairs per chip, avoiding the use of sequential barcodes on the same chip.

### Reverse Transcription Quantitative Real-Time PCR (RT-qPCR)

RNA was isolated from the hippocampi of 15-month-old wt and tg mice (n=6 pairs), using the mirVana™ PARIS™ RNA isolation kit (Invitrogen; Cat #AM1556). RNA sample concentrations and integrity were determined using spectrophotometry (Nanodrop 1000; Thermo Scientific, USA) and a Bioanalyzer, using an RNA 6000 NanoLabchip (Bioanalyzer 2100; Agilent Technologies, USA). Only samples with an average RNA integrity number >9 were used. Gene-specific complementary DNA (cDNA) libraries were prepared using the TaqMan mRNA reverse transcription kit (Applied Biosystems), primed using random hexamers, according to manufacturer’s instructions. Amplification was performed in triplicate using 20x TaqMan Gene Expression Assays, with *Hprt* as housekeeping (primers used; *Trem2*–04209424_m1, *Tyrobp*–00449152_m1, *Cst7*–00438351_m1, *Hprt*–03024075_m1). These reactions were amplified using a ViiA7 Real-Time PCR System (Applied Biosystems). Data were normalised against *Hprt* expression, and the 2^-ΔΔCT^ method was used to calculate fold-change. A two-tailed paired Student’s *T*-test was then used to determine statistically significant changes with a threshold of *p* < 0.05.

### Bioinformatics

Data were initially analysed as described previously [33]. Briefly, data from each barcoded library were separated into data files on the Ion Torrent Suite version 5.4 (Life Technologies, USA). Adapter sequences were trimmed using AdapterRemoval v2.1.7, and reads were trimmed for quality using Trimmomatic v0.38. Reads were aligned to the *Mus* *musculus* GRCm38.95 reference genome using STAR v2.5.4b. Additionally, miRNA reads were extracted and analysed separately using miRDeep2 v0.1.2, and piRNA data were obtained from piRNABank. Data were analysed using R version 4.0.2 in RStudio v1.3.959. The function *featureCounts* from the package *Rsubread* generated raw count data. Differential expression analysis was performed using the *exactTest* function from the package *edgeR*. Heatmaps and agglomerative hierarchical clustering were achieved using the package *pheatmap*.

For mRNA, differentially expressed genes were entered into the web interface of the Search Tool for the Retrieval of Interacting Genes/Proteins (STRING) [34–44], which is a curated database of experimentally validated protein-protein interactions and co-expression. STRING-db was also used to perform Markov clustering (MCL; a form of unsupervised graph clustering that shows robust behaviour in the analysis of protein-protein interactions), using an inflation parameter of 1.8, a value shown to be optimal in these kinds of analyses [45, 46]. The purpose of this was to discover related clusters and nodes within the protein network. Clusters grouped by STRING were entered into *Enrichr* [47, 48], a web interface for functional enrichment of gene lists, using the database Wikipathways. Transcription factor enrichment analysis was performed using the webserver BART (Binding Analysis for Regulation of Transcription) [49, 50] for mRNA.

MiRNA targets were explored using DIANA Tarbase v.8 [51], which lists experimentally validated microRNA-target interactions from a variety of sources. Additionally, transcription factors targeting miRNA loci were found using TransmiR [52, 53].

### Data Availability

The data discussed here have been deposited in NCBI’s Gene Expression Omnibus [54] and are accessible through GEO Series accession number GSE163878 (https://www.ncbi.nlm.nih.gov/geo/query/acc.cgi?acc=GSE163878).

## Results

We aimed to understand the extent of transcriptomic change between 15-month APP/PS1 mice and wt, an age where extensive plaque burden is routinely observed [32]. To achieve this, we identified the RNA biotypes represented within differentially expressed gene set. To explore the biological impact of the differentially expressed genes, we used a bioinformatics approach and we explored possible interactions between different RNA species within the dataset, drawing on the strength of the simultaneous whole-transcriptome analysis.

### RNA-Seq Reveals Genes Differentially Expressed Between APP/PS1 and Wild-Type Control Mice at 15 Months

Here, we provide a holistic understanding of transcriptomic changes in the APP/PS1 model of AD by analysis of differentially expressed genes using our newly developed protocol [33] which allows access to all RNA biotypes. Following false discovery rate correction (FDR < 0.05; Table 1), 20 genes were significantly changed in the APP/PS1 mice including both *App* and *Psen1* (log fold change 1.22 and 0.85 respectively; edgeR package for R) as expected. We also found a number of other genes commonly found differentially expressed in AD and involved in disrupted processes, such as *Trem2* (log fold change 1.35), *Tyrobp* (log FC 2.21), and *Gfap* (log FC 1.66).

**Table 1:** Differentially expressed genes between APP/PS1 mice and wild-type controls (15 months), at FDR < 0.05. Note Lamr1-ps1 (Laminin receptor 1 pseudogene 1) is believed to be an artifact of transgene insertion in this model and was therefore excluded from further analyses. Asterisks indicate genes associated with microglial function by literature search

Ensembl ID	Gene symbol	log2 FC	log2 CPM	*p*-value	FDR
ENSMUSG00000081229	*Lamr1-ps1*	6.729103	1.044629	3.38E-19	6.69E-15
ENSMUSG00000046805	Mpeg1*	1.990667	4.700738	2.06E-17	2.04E-13
ENSMUSG00000030789	Itgax*	3.608	1.839671	8.00E-13	5.28E-09
ENSMUSG00000068129	Cst7*	3.439047	1.547066	7.28E-12	3.60E-08
ENSMUSG00000079037	*Prnp*	1.549274	8.957976	7.77E-11	3.08E-07
ENSMUSG00000079293	Clec7a*	3.198116	0.821581	4.42E-10	1.46E-06
ENSMUSG00000018927	Ccl6*	3.39885	0.393266	1.02E-08	2.89E-05
ENSMUSG00000023992	Trem2*	1.354341	3.074181	2.30E-07	0.000569
ENSMUSG00000030579	Tyrobp*	2.208173	2.011728	1.55E-06	0.003405
ENSMUSG00000022892	*App*	1.225	10.43842	1.83E-06	0.00363
ENSMUSG00000069516	Lyz2*	2.174822	3.03749	2.57E-06	0.004391
ENSMUSG00000004707	Ly9*	2.23793	0.262328	2.66E-06	0.004391
ENSMUSG00000036896	C1qc*	1.999691	4.110238	2.99E-06	0.004544
ENSMUSG00000040552	C3ar1*	1.680807	1.26491	3.48E-06	0.004915
ENSMUSG00000069515	Lyz1*	2.629014	1.000814	4.05E-06	0.005342
ENSMUSG00000073418	C4b*	1.42005	7.102349	1.07E-05	0.013281
ENSMUSG00000019969	*Psen1*	0.848874	5.434479	1.20E-05	0.013978
ENSMUSG00000015451	C4a*	1.441767	5.741604	2.20E-05	0.024169
ENSMUSG00000027015	*Cybrd1*	1.999986	1.791556	2.69E-05	0.027984
ENSMUSG00000020932	*Gfap*	1.658875	8.593936	4.30E-05	0.042537

**Fig. 1:**
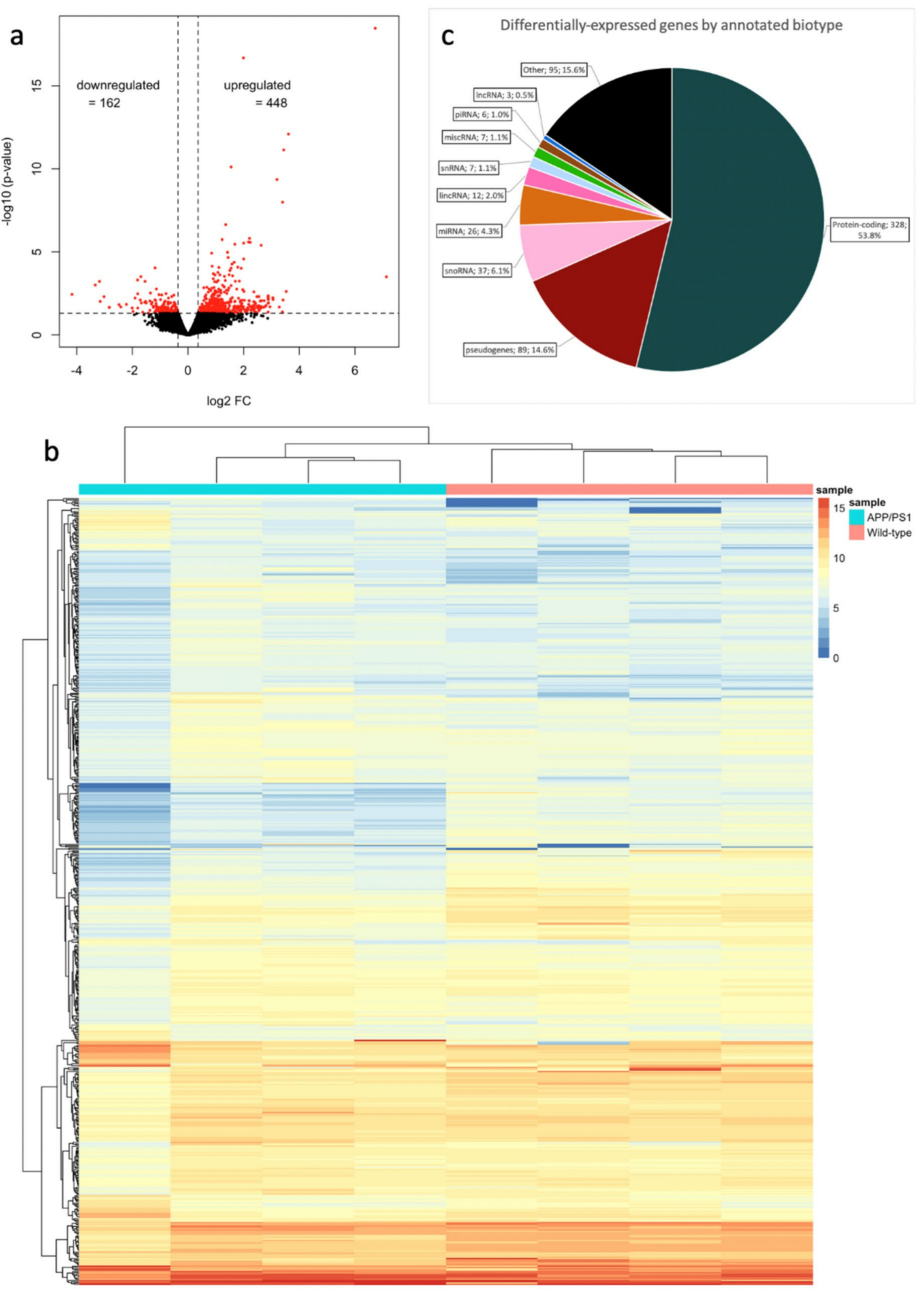
Differential gene expression between 15-month APP/PS1 and wild-type control mice. **a** Volcano plot of identified genes, using *p* < 0.05 and log2FC > 0.359 and < –0.359 as cutoff. differentially expressed genes shown in red. **b** Heatmap showing log2 counts-per-million (CPM) of all 610 differentially expressed genes. Rows and columns are clustered using hierarchical clustering. **c** Pie chart showing relative proportion of differentially expressed genes divided into biotypes annotated in the GRCm38.95 genome. Each label shows biotype, raw number, and percentage

Lower stringency in data analysis can discover important genes for further investigation that would otherwise be filtered out by more stringent settings. Therefore, we made use of unadjusted *p*-values for further analyses of these data. At unadjusted *p* < 0.05, the list of differentially expressed genes expanded to 610 genes, allowing for a much more in-depth analysis of gene expression. The full list of differentially expressed genes is shown in Online Resource 1. Of these 610 genes, 448 are upregulated and 162 downregulated (log2 fold-change > 0.359 and < –0.359; Fig. 1a).

Next, to analyse relationships and similarities between samples and genes, hierarchical clustering was performed. Wt and tg samples clustered to type, indicating that their gene expression profiles are more similar within-group than between-groups (Fig. 1b). This suggests that differentially expressed genes can clearly distinguish tg from wt animals; supporting the validity of the results.

### Validation of RNA-Seq Data by RT-qPCR

In order to further validate the data obtained from the modified RNA-Seq protocol further, the expression of selected genes as measured by RNA-Seq was compared to RT-qPCR measurement. For this analysis, we chose the *Cst7* as the gene with one of the highest fold-change values, and *Trem2* and *Tyrobp* as genes commonly identified in literature. All three of these genes were also found differentially expressed by RT-qPCR (Online Resource 2).

### Functional Enrichment Analysis Highlights Key Gene Clusters and Pathways Changed in the APP/PS1 Mouse Model of AD

**Fig. 2:**
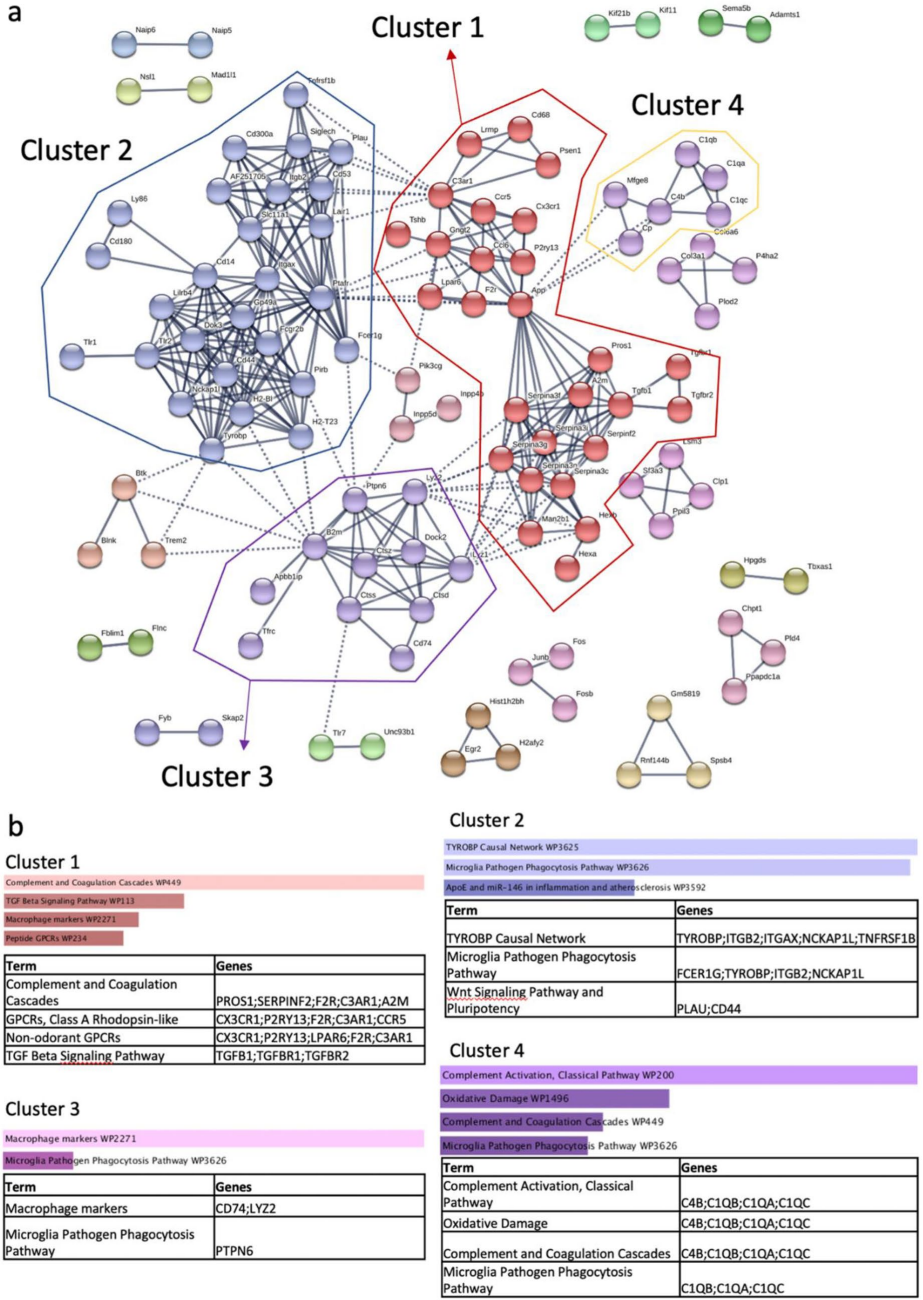
Clustering and functional annotation of differentially expressed mRNA. **a** STRING Markov clustering of protein-protein interactions using an inflation parameter of 1.8. Separate clusters are outlined and labelled. **b** *Enrichr* functional annotation of the clusters formed in (**a**) by Mouse Wikipathways, sorted by combined score and colour coded. Only significantly enriched pathways (*p* < 0.05) are shown

To better understand enriched processes and pathways among differentially expressed protein-coding genes, Markov clustering of differentially expressed mRNA in STRING was used to analyse protein-protein interactions. An inflation parameter of 1.8 was used, and only experimentally validated relationships, curated databases, and conserved protein co-expression were considered. This method grouped differentially expressed protein-coding genes into four major clusters based on their protein interactions (Fig. 2a). Each cluster had unique strongly enriched pathways, as shown in Fig. 6b, with an overriding theme of microglia involvement and complement activation. Cluster 1 was enriched for the complement and coagulation cascade pathway, Cluster 2 for the TYROBP causal network (a pathway regulating signal transduction across microglial plasma membranes in disease-associated microglia [DAM]), Cluster 3 for Macrophage markers, and Cluster 4 for Classical complement activation. Indeed, 14 of the top 20 differentially expressed mRNA are associated with microglia indicating widespread changes in microglial function are occurring in the APP/PS1 mouse by 15 months (identified by asterisks in Table 1).

**Fig. 3:**
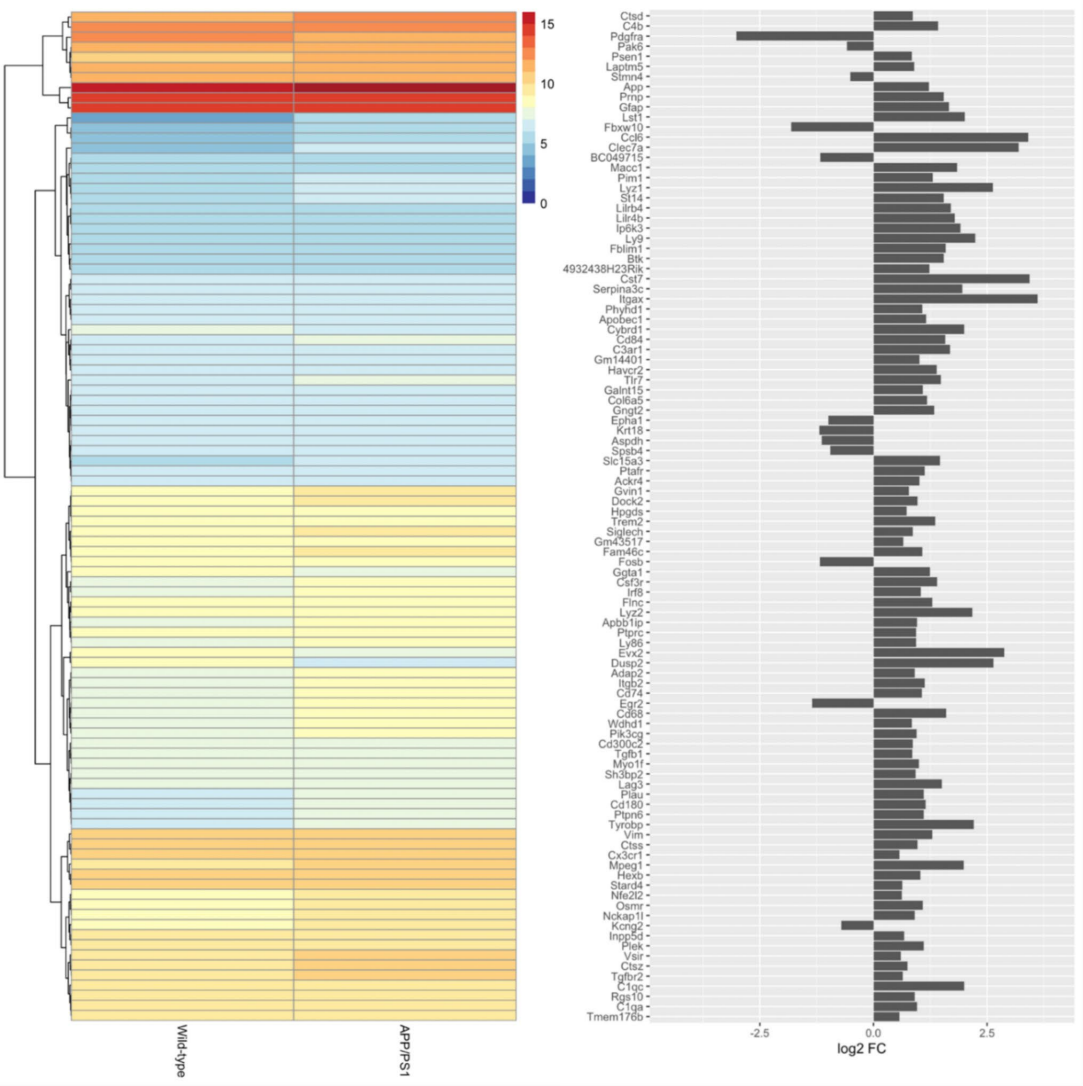
Differential expression of protein-coding genes between 15-month-old APP/PS1 and wild-type control mice. The heatmap shows the mean log2 CPM of the top 100 differentially expressed protein-coding genes, averaged across samples for each condition, as annotated in the *Mus* *musculus* GRCm38.95 reference genome. The dendrogram represents hierarchical clustering of genes based on mean normalized expression. Each row of the heatmap corresponds to the gene symbols as shown in the bar chart (left), which indicates log2 fold-change of each gene

Further exploration of these clusters was performed by functional enrichment and pathway analysis using *Enrichr*. Interestingly, Cluster 1 contained the genes encoding TGF-β1 and its two major receptors (*Tgfb1*, *Tgfbr1* and *Tgfbr2*). All three genes were upregulated in APP/PS1 mice (log FC 0.86, 0.51, 0.65; p = 0.004, 0.01, 0.004 respectively; Fig. 2a, b and Fig. 3). TGF-β1 is a cytokine preferentially secreted by astrocytes in response to inflammatory conditions, acting to modulate microglial reactivity [55]. Human AD patients show an increased expression of TGF-β1, but a reduction in its anti-inflammatory efficacy through a reduction in the expression of its receptors [56, 57]. Such an increase has not previously been reported in APP/PS1 mice, though the concomitant increase in the expression of *Tgfbr1* and *Tgfbr2* points to this being a response to, and attempt to mediate, microglial reactivity and rampant neuroinflammation.

### Analysis of Enriched Transcription Factors

Next, we aimed to understand the underlying mechanisms influencing the differentially expressed genes. Utilizing the webserver BART (binding analysis for regulation of transcription) [49, 50], which draws on ChIP-Seq (chromatin immunoprecipitation sequencing) data, we identified key regulators of gene expression through detection of shared and conserved regulatory elements within our 610-gene input list and cross-referenced these with the differentially expressed mRNA.

Using this approach, we found six differentially expressed transcriptional regulators (Table 2; refer to Online Resource 3 for a full list of predicted transcription factors). All six of these transcription factors have been previously tied to microglial function, apoptosis, or autophagy [58–63]. These results suggest that these six regulatory factors may act as major regulatory hubs and underlie many of the changed pathways seen in this gene set.

**Table 2:** Differentially expressed transcriptional regulators that target differentially expressed genes, as predicted by BART, and their biological relevance

Transcription factor	Wilcoxon statistic	Wilcoxon *p*-value	Z-score	Max AUC	Biological relevance
IRF8	7.147	4.43E-13	2.328	0.843	Activates microglia [61]
JUNB	6.328	1.24E-10	2.728	0.874	Inhibits apoptosis [59]
c-FOS	4.466	3.98E-06	2.702	0.737	Promotes apoptosis [60]
LMO2	2.712	3.34E-03	3.736	0.654	Activates microglia [58]
RUNX1	2.293	1.09E-02	0.416	0.656	Toll-like receptor signalling in microglia [63]
NFE2L2	2.122	1.69E-02	1.822	0.702	Regulates autophagy [62]

### Biotype Analysis of RNA-Seq Data

**Fig. 4:**
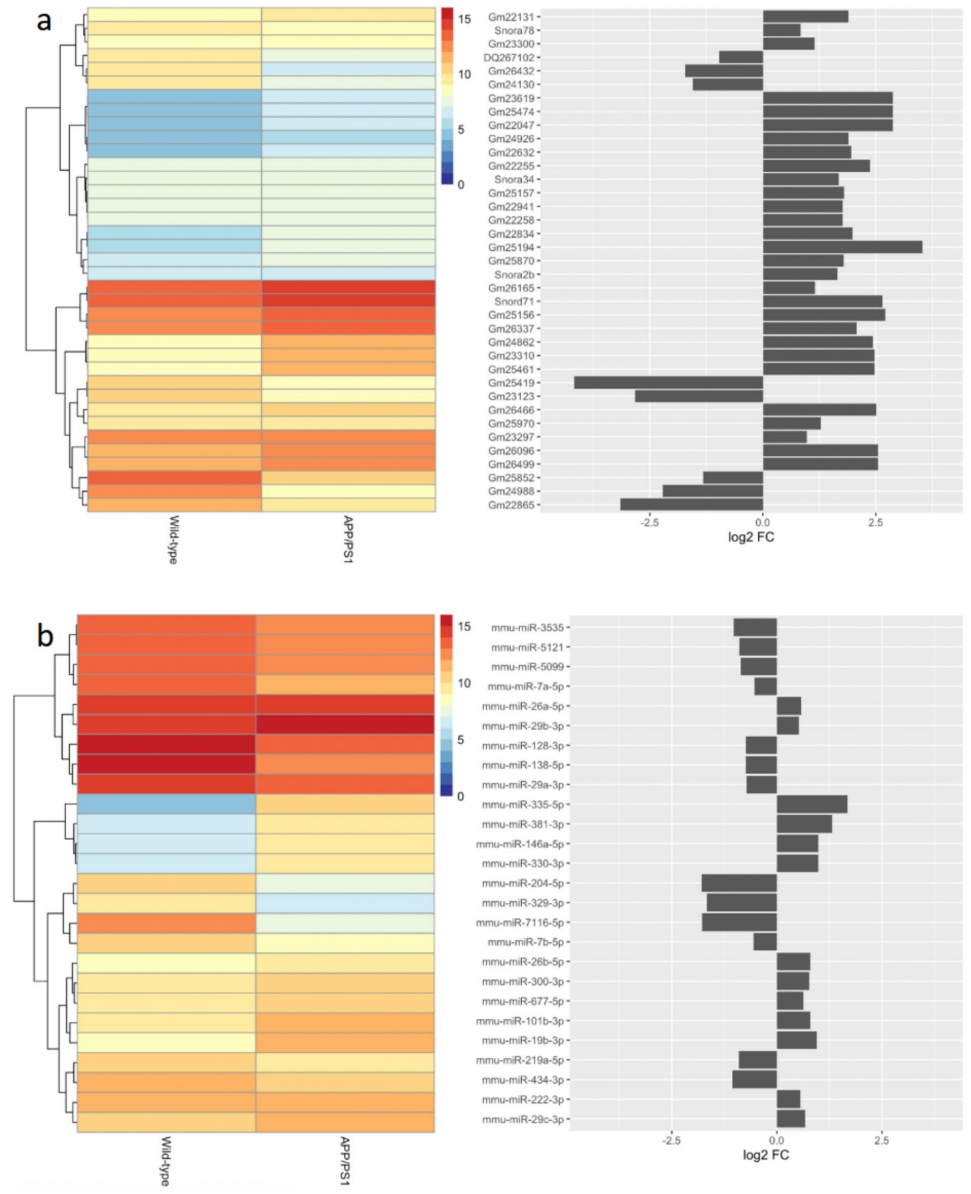
Differential expression of snoRNA (**a**) and miRNA (**b**) between 15-month-old APP/PS1 and wild-type control mice. The heatmaps show the mean log2 CPM of differentially expressed snoRNA (**a**) and miRNA (**b**), averaged across samples for each condition, as annotated in the *Mus* *musculus* GRCm38.95 reference genome. The dendrograms represent the hierarchical clustering of genes based on mean normalised expression. Each row of the heatmaps corresponds to the gene symbols as shown in the bar charts (left), which indicates log2 fold-change of each transcript

**Fig. 5:**
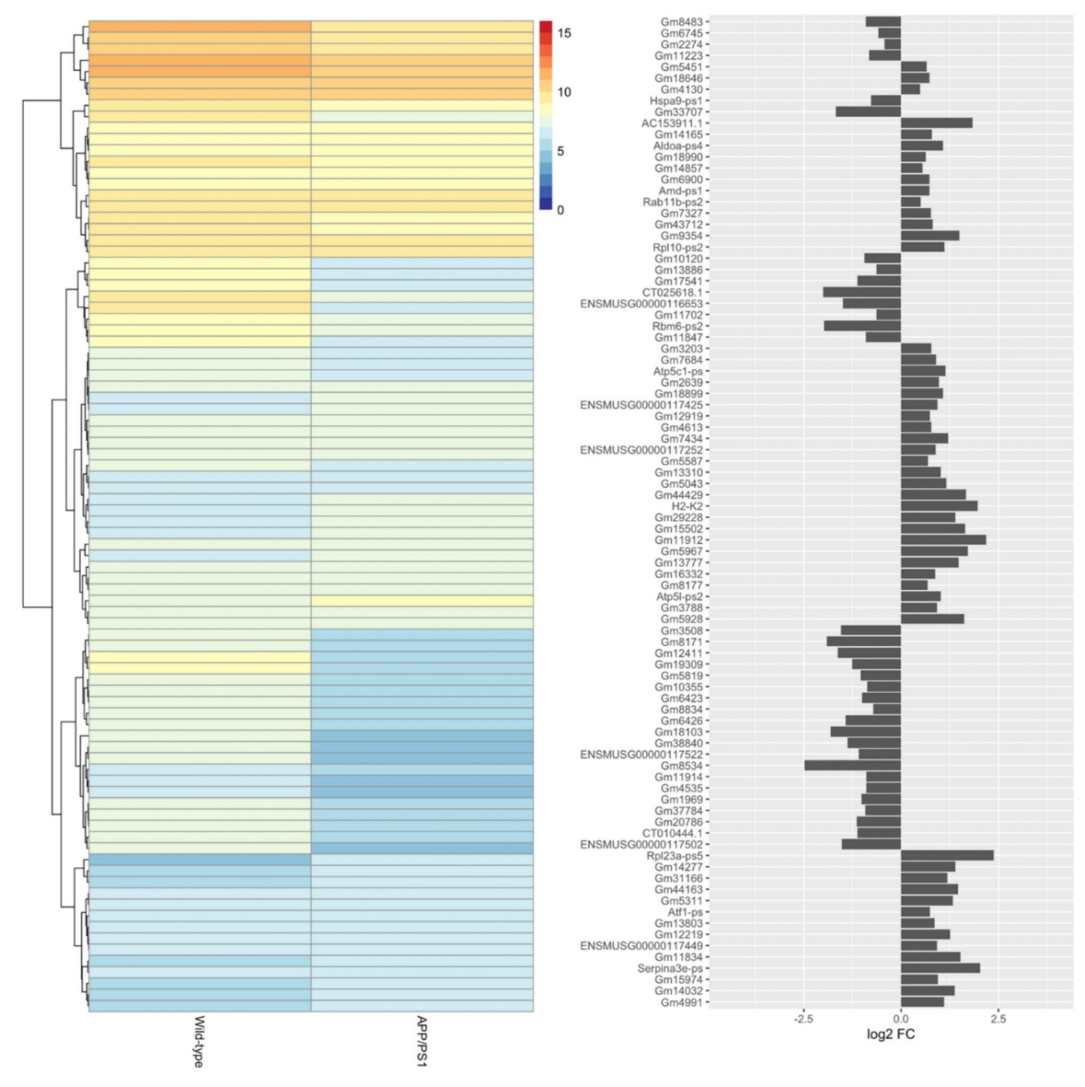
Differential expression of pseudogenes between 15-month-old APP/PS1 and wild-type control mice. The heatmap shows the mean log2 CPM of differentially expressed pseudogenes, averaged across samples for each condition, as annotated in the *Mus* *musculus* GRCm38.95 reference genome. The dendrogram represents the hierarchical clustering of genes based on the mean normalised expression. Each row of the heatmap corresponds to the gene symbols as shown in the bar chart (left), which indicates log2 fold-change of each gene

**Fig. 6:**
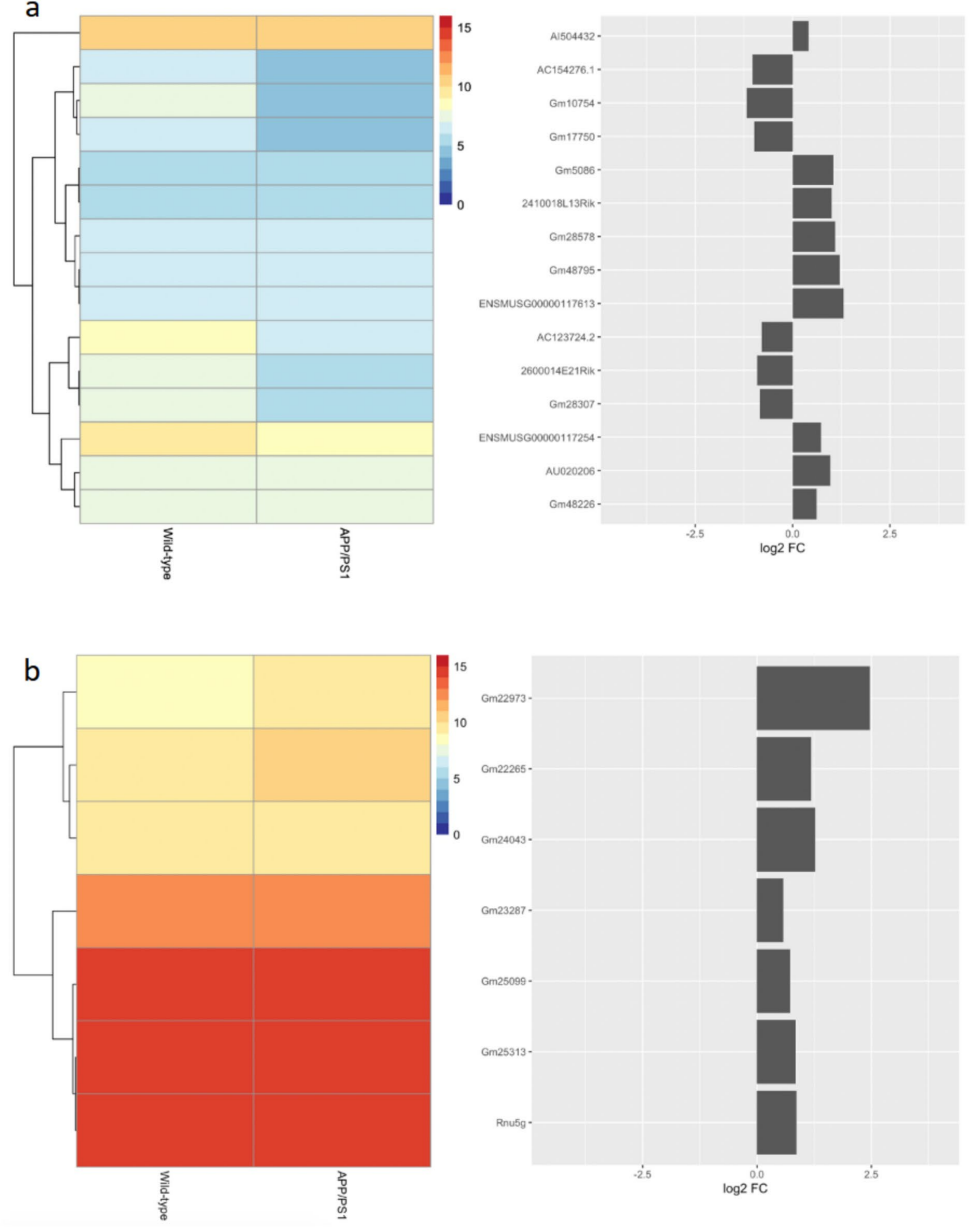
Differential expression of lncRNA (**a**) and snRNA (**b**) between 15-month-old APP/PS1 and wild-type control mice. The heatmaps show the mean log2 CPM of differentially expressed lncRNA (**a**) and snRNA (**b**), averaged across samples for each condition, as annotated in the *Mus* *musculus* GRCm38.95 reference genome. The dendrograms represent the hierarchical clustering of genes based on mean normalised expression. Each row of the heatmaps corresponds to the gene symbols as shown in the bar charts (left), which indicates log2 fold-change of each transcript

To understand the extent of transcriptomic change within the APP/PS1 mouse model, we identified the RNA biotypes represented within the differentially expressed gene set. This analysis showed that 328 of the differentially expressed species were derived from protein-coding genes, 89 pseudogenes, 37 snoRNA, 26 miRNA, 12 long intergenic non-coding RNA (lincRNA), seven snRNA, seven non-classified RNA (miscRNA), six piRNA, and three lncRNA (GRCm38.100 reference genome; mirDeep2.0; piRNABank; Figures 1c, 3, 4, 5, and 6). The proportions of protein-coding to non-coding gene biotypes were consistent with reported cellular RNA contents [64], and these were higher and more diverse than other data obtained from more conventional RNA-Seq library construction techniques using similar samples [33, 65, 66].

### Functional Enrichment Analysis of Non-coding RNA

miRNA: We found that changes in miRNA expression echo functional enrichment seen in mRNA expression. Of the 26 miRNA differentially expressed in APP/PS1 mice, 13 were found to be upregulated, and 13 downregulated (Fig. 4b). Exploring the functions of these miRNA by literature search reveals 19 miRNA tied to microglial function (Table 3). MicroRNA involved in inhibiting microglial activation were significantly downregulated—for example, miR-204-5p has been shown to suppress microglial activation (log2 FC = −1.79, *p* < 0.0001 [67]), miR-7116-5p similarly inhibits microglial activation by targeting TNF-α, which is necessary for pro-inflammatory microglial responses (log2 FC = –1.78, *p* < 0.0001 [68]), and miR-7a-5p suppresses microglial inflammatory activation (log2 FC = –0.53, *p* < 0.05 [69]). Conversely, several miRNAs were upregulated that have been shown to have pro-inflammatory or pro-apoptotic functions and were expressed in or associated with microglia, including miR-146a-5p (log2 FC = 0.98, *p* < 0.01) and miR-29b-3p (log2 FC = 0.52, *p* < 0.05) [70, 71]. Overall, we see upregulation of pro-inflammatory and pro-apoptotic miRNA, and downregulation of anti-inflammatory and anti-apoptotic miRNA, mirroring the functions of upregulated protein-coding genes.

**Table 3:** Differentially expressed miRNA related to microglial function and their functional significance

MicroRNA	log2 FC	*p*-value	Function	Reference
mmu-miR-335-5p	1.68	< 0.0001	Expressed in microglia; anti-inflammatory.	[77]
mmu-miR-381-3p	1.32	< 0.005	Expressed in microglia; modulates NF-κB signalling.	[78]
mmu-miR-330-3p	0.99	< 0.01	Expressed in microglia; modulates NF-κB signalling.	[79]
mmu-miR-146a-5p	0.98	< 0.01	Expressed in microglia; Primes microglia for activation.	[70]
mmu-miR-19b-3p	0.96	< 0.005	Expressed in microglia; increases production of inflammatory cytokines like TNF-α.	[80]
mmu-miR-101b-3p	0.79	< 0.005	Expressed in microglia; pro-inflammatory, pro-pyroptotic.	[81]
mmu-miR-26b-5p	0.79	< 0.05	Expressed in microglia; anti-apoptotic through targeting IL-6.	[82]
mmu-miR-29c-3p	0.68	< 0.05	Expressed in microglia; anti-inflammatory effects.	[83]
mmu-miR-26a-5p	0.58	< 0.01	Expressed in microglia; anti-inflammatory, modulates TNF-α.	[84]
mmu-miR-222-3p	0.55	< 0.05	Expressed in microglia; pro-inflammatory, pro-apoptotic.	[85]
mmu-miR-29b-3p	0.52	< 0.05	Expressed in microglia; Promotes expression of pro-inflammatory cytokines.	[71]
mmu-miR-7a-5p	−0.53	< 0.05	Suppresses microglial activation.	[23]
mmu-miR-138-5p	−0.73	< 0.0001	Expressed in microglia; anti-inflammatory, anti-apoptotic, Caspase-1.	[86]
mmu-miR-29a-3p	−0.73	< 0.0001	Suppresses microglial activation.	[87]
mmu-miR-128-3p	−0.74	< 0.0001	Suppresses microglial activation.	[88]
mmu-miR-5121	−0.89	< 0.0001	Released from microglia in exosomes–promotes neuronal repair.	[89]
mmu-miR-329-3p	−1.66	< 0.05	Promotes microglial activation; pro-apoptotic.	[90]
mmu-miR-7116-5p	−1.78	< 0.0001	Inhibits TNF-α expression; suppresses microglial activation.	[68]
mmu-miR-204-5p	−1.79	< 0.0001	Suppresses microglial activation.	[67]

Pseudogenes: Intriguingly, pseudogenes make up the second largest group of differentially expressed RNA, with 88 annotated genes (54 upregulated, 34 downregulated). Unfortunately, little information is available as to the functional relevance of these transcripts (Fig. 5), though this is likely due to the paucity of information about functional pseudogenes in general. Only very recently has research into pseudogenes begun to unearth what roles they may play. Various pseudogenes have been identified as differentially expressed in human and animal models of AD [72–74], and as our understanding of pseudogene functionality increases, it would be useful to revisit the pseudogenes identified in this analysis.

snoRNA: Besides pseudogenes, snoRNA represents the largest proportion of differentially expressed ncRNA in this dataset (Fig. 4a). Of these, 8 were downregulated and 29 upregulated, although only 4 of these have distinct snoRNA classifications—*Snora78* (log2 FC = 0.83; *p* < 0.01); *Snora34* (log2 FC = 1.68; *p* < 0.05); *Snora2b* (log2 FC = 1.65; *p* < 0.05); and *Snord71* (log2 FC = 2.65; *p* < 0.05). *Snora34* (ACA34) and *Snora2b* (ACA2b) are predicted to guide pseudouridylation of 28S rRNA, *Snora78* (ACA64) is predicted to be involved in pseudouridylation of 5.8S rRNA, while *Snord71* (MBII-239) guides methylation of 5.8S rRNA. Curiously, some evidence suggests that 28S rRNA is affected in APP/PS1 mice [75], and that there may be an excess of pseudouridine in AD patients [76]. The effect of changes in snoRNA expression and resulting rRNA modifications is still unclear. However, given the crucial role rRNA modifications play in downstream gene expression through mRNA selection, these changes may have far-reaching downstream influences on AD-like pathology.

lncRNA: When exploring the functionality of the 15 combined lncRNA and lincRNA (Fig. 6a), two specific lncRNA stand out—AU020206 (log2 FC = 0.96; *p* < 0.05) and 2410018L13Rik (log2 FC = 1.00; *p* < 0.05). AU020206 has been implicated in cholesterol homeostasis and is differentially expressed in atherosclerosis [91, 92]. Cholesterol homeostasis has a complex relationship with AD, with interactions shown between ApoE4, Aβ, and cholesterol [93, 94]. 2410018L13Rik, meanwhile, is a lncRNA associated with ageing [95]. The other differentially expressed lncRNA in our dataset are poorly characterised or understood.

snRNA: Of the 7 reported differentially expressed snRNA, all of which were found to be upregulated (Fig. 6b), only 1 has specific classification—*Rnu5g*, coding for the U5 snRNA (log2 FC = 0.86; *p* < 0.05). U5 snRNA is unique in that it is a component of both the major and minor spliceosome. Spliceosomal components have been found to aggregate in close proximity to Aβ plaques in AD brains, suggesting some kind of relationship between AD pathology and the spliceosome [96]. Alternative splicing can play a major role in the development of AD phenotypes through alterations in the exon combinations for a number of key genes [28, 97]. Additionally, the minor spliceosome has recently been implicated in neurodegenerative disorders—notably spinal muscular atrophy (SMA) and amyotrophic lateral sclerosis (ALS) [98]. The functional significance of the minor spliceosome is relatively poorly understood, and its potential roles in AD have not been fully investigated.

### A Synthesis of RNA Expression—Investigating RNA-RNA Interactions

While much can be learned from individual analysis of differentially expressed gene biotypes, one major strength of simultaneous whole-transcriptome analysis comes from the ability to identify possible interactions between different RNA species within the same dataset.

miRNA and Their mRNA Targets: The primary example of RNA-RNA interactions is arguably between miRNA and their mRNA targets—a relationship that is among the more well-understood and well-characterised. Information about miRNA:mRNA relationships within our differentially expressed genesets were obtained using DIANA-Tarbase v.8 [51], which contains over 300,000 mouse miRNA-mRNA interactions, and miRWalk 2.0, which contains ~2000 mouse miRNA and 29,000 mRNA.

Analysis of the top differentially expressed protein-coding genes from the RNA-Seq dataset revealed a total of 60 interactions between 15 miRNA and 42 mRNA (Table 4.). Most of these interactions show directionality consistent with the transcriptional repression effect of miRNA—11 of 21 mRNA targets of upregulated miRNA are themselves downregulated, and 35 of 42 mRNA targets of downregulated miRNA are upregulated.

**Table 4.**
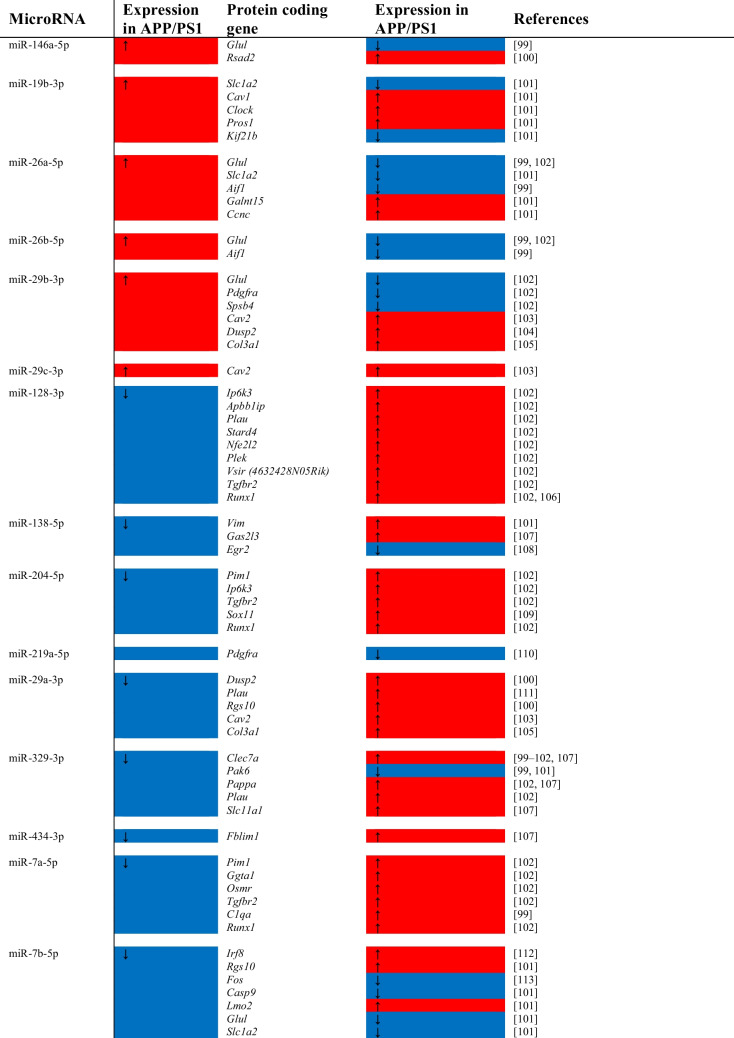
Differentially expressed miRNA and their validated targets are also differentially expressed in the RNA-Seq data. Targets combined from DIANA TarBase v.8 and miRWalk 2.0

Transcription Factors and miRNA Targets: These data also allow for the investigation of how transcription factors may affect the expression of non-coding RNA. This kind of analysis is most accessible for miRNA, whose expression, genetic location, and promoter activity are more well-characterised than other ncRNA species. To explore the relationship between transcription factors and miRNA, genes associated with the Gene Ontology keyword “transcription factor activity” were entered into TransmiR, a curated database of transcription factor-miRNA interactions [52, 53]. This analysis resulted in finding 58 total interactions of 8 unique transcription factors (differentially expressed in these APP/PS1 samples) and 18 miRNA (Table 5). Of these 58 interactions, in 29 the miRNA expression is changed in the same direction as the transcription factor.

**Table 5.**
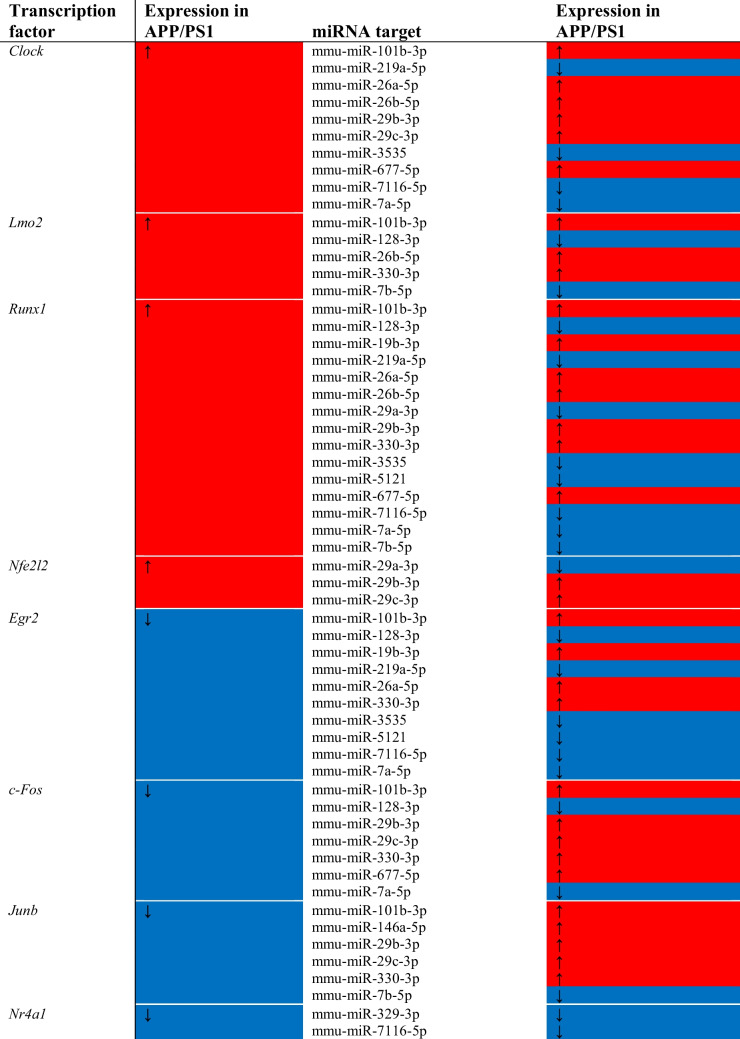
Differentially expressed transcription factors and their differentially expressed miRNA targets from the RNA-Seq data, as noted in the TransmiR database

Echoing their importance to transcriptional regulation of protein-coding genes, the transcription factors *c-Fos*, *Junb*, *Lmo2*, *Nfe2l2*, and *Runx1* are the key regulatory factors controlling miRNA expression in this model (Table 5). The other transcription factors seen in this analysis are *Clock* (Circadian Locomotor Output Cycles Kaput), *Egr2* (Early growth response protein 2), and *Nr4a1* (Nuclear receptor 4A1).

Altogether, these links reinforce the changes in gene expression seen in APP/PS1 mice at 15 months. The transcription factors *Runx1*, *c-Fos*, *Junb*, *Nfe2l2*, and *Lmo2* seem to be hubs of transcriptional regulation, while *Clock*, *Egr2*, and *Nr4a1* add an additional factor of miRNA regulation to the equation. These transcription factors and their regulated miRNA, coupled with the gene targets of those miRNA, provide a picture of a system undergoing widespread pathological activation of microglia and astrocytes, gliosis, and neuroinflammation, as well as processes attempting to mediate the negative effects of such inflammation.

## Discussion

### The Aberrant Transcriptome of APP/PS1 Mice

Understanding the molecular complexity underpinning AD is essential for the development of disease-modifying therapies. APP/PS1 mice exhibit synaptic dysfunction, synaptic and neuronal loss, as well as plaque formation and thus provide a useful tool to study the early stages of AD. The ability of the whole-transcriptome RNA-Seq protocol utilised in this study to analyse complex RNA-RNA interactions offers a unique approach to RNA-Seq data. Using whole-transcriptome RNA-Seq to encapsulate coding and non-coding transcripts, we have provided a unique snapshot of alterations in the hippocampal transcriptome of 15-month-old APP/PS1 mice, identifying key control hubs which may be potential novel therapeutic targets.

Functional enrichment analysis of differentially expressed protein-coding genes was indicative of the widespread activation of microglia and astrocytes. Activation of microglia from their “resting”, homeostatic state to a pro-inflammatory, “reactive” state is a key component of the brain’s response to injury. This activation results in significant changes to the morphology and transcriptome of the cells [114]. It is considered a key driver of AD pathology [115–117]. Alongside this, we found reduced expression of the astrocyte genes *Glul*, *Slc1a2*, and *Slc1a3*, which are necessary for glutamate metabolism in “normal”. These changes are suggestive of a shift away from healthy astrocyte function towards pathological astrocyte activation. Similar impairment of glutamate metabolism is commonly observed in astrocytes in both mouse models of AD and human AD patients [118–120].

Analysis of enriched transcription factors revealed the transcriptional control hubs related to changes in glial cells. Eight transcription factors—AP-1 (as *c-Fos* and *Junb*), *Clock*, *Egr2*, *Irf8*, *Lmo2*, *Nr4a1*, *Nfe2l2*, and *Runx1*—seem to underlie most of the gene expression changes observed. *Clock*, *Egr2*, *Irf8*, *Lmo2*, *Nr4a1*, *Nfe2l2*, and *Runx1* are all intimately tied to glial function. The results of our transcription factor analysis consistently show downregulation of transcription factors mediating normal/healthy glial function (*Egr2*, *Nr4a1*) and upregulation of transcription factors guiding reactivity of glia (*Irf8*, *Lmo2*, *Nfe2l2*, and *Runx1*). These effects are mirrored in the functional analysis of the miRNA targets of the identified transcription factors, with *Egr2* and *Nr4a1* regulating expression of several miRNA that suppress microglial activation (miR-128-3p, miR-329-3p, miR-5121, miR-7116-5p, miR-7a-5p), while upregulated transcription factors target miRNA that themselves promote microglial activation and inflammation (e.g. miR-101b-3p, miR-26a-5p, miR-26b-5p, miR-330-3p). These findings highlight potential alternative approaches to therapeutics for AD that could target these genes/proteins, or upstream/downstream pathways. In particular, targeting pathways that are known to be dysregulated in AD—such as TGF-β signalling, Toll-like receptor signalling, or more broadly excessive microglial and astrocyte activation and gliosis—but that are currently not being explored in favour of Amyloid-targeting therapies may prove a more fruitful approach to therapeutics given the difficulties in finding effective Amyloid-targeting therapies.

#### Limitations

One hurdle to a thorough analysis of such in-depth RNA-Seq data is the *status quo* of our understanding of the mouse transcriptome. While great strides have been made in identifying, cataloguing, and annotating the various species of non-coding RNA, there remain significant gaps, both in incomplete annotation and knowledge of functional significance. With the RNA-Seq protocol used in this study following a global transcriptome approach, not selecting for specific types of RNA, many differentially expressed RNA were identified that have not yet been annotated (“predicted genes”). Even many genes that have been named have not been functionally explored. While some biotypes of RNA like messenger RNA and miRNA are relatively well-characterised, the workings of lncRNA, piRNA, and pseudogenes are yet to be understood. Furthermore, as of yet, no unified database exists for functional analysis of all biotypes of ncRNA. This makes it difficult to analyse whole-transcriptome data, necessitating lengthy and detailed manual literature searches. However, new databases are in constant development, be they curated or predictive, and such developments will improve the ability to perform functional analysis of large-scale RNA-Seq data.

The APP/PS1 mouse mutations reflect those observed in the rare familial form of AD but lack several features common to sporadic AD in humans, including tau pathology. Further, using this model, we have chosen to assess gene expression of all neural cell types simultaneously and not sort by phenotype. Performing single-cell or single-nucleus RNA-seq from this tissue would corroborate the extent to which microglia themselves are altered and validate the findings described here. Despite this, our results portrayed far more complex and widespread pathological processes than amyloidopathy, with many overlaps with transcriptome changes seen in humans with AD, particularly with regard to microglial complement activation [121–125].

## Conclusions

Our research highlights the potential of moving away from solely developing amyloid-targeting therapies and instead moving towards multifactorial therapies that function more effectively than the sum of their parts. It is clear that runaway inflammation and reactive gliosis are key components to the later stages of AD and should thus be considered potential targets for symptom alleviation-focused treatments. However, such targets may also be useful for developing prevention-focused treatments, since there is some evidence that the use of anti-inflammatory drugs may affect the incidence as well as the severity of AD in humans [126, 127]. Without ignoring the contribution of amyloidosis to AD pathology, combination therapies targeting both reduction of amyloid load and additional up- or down-stream processes may serve as better treatments in the short term [128, 129].

In this study, we show that a whole-transcriptome RNA sequencing approach can identify gene expression changes of both coding and non-coding genes in a murine model of late-stage AD. This approach reduces additional biases from separation and purification of individual RNA subtypes. To our knowledge, this is the first such exploration of the transcriptome in 15-month-old APP/PS1 mice, and these data show widespread dysfunction of immune processes and dysregulation of microglia- and astrocyte-specific pathways. This research also identifies networks of potential RNA-RNA interactions and predicts altered transcription factors driving and underlying these changes. These findings may open doors to therapeutic interventions that target either these genes/proteins or upstream/downstream pathways.

## Supplementary Information

Below is the link to the electronic supplementary material.

Supplementary file1 (CSV 35 KB)

Supplementary file2 (PDF 75 KB)

Supplementary file3 (CSV 4 KB)

## Data Availability

The data discussed here have been deposited in NCBI’s Gene Expression Omnibus [54] and are accessible through GEO Series accession number GSE163878 (https://www.ncbi.nlm.nih.gov/geo/query/acc.cgi?acc=GSE163878).
