# Supplementary material for: Whole Transcriptome RNA-Seq Reveals Drivers of Pathological Dysfunction in a Transgenic Model of Alzheimer’s Disease
Source: Mol Neurobiol. 2025 Apr 5;62(8):10144–64. doi: 10.1007/s12035-025-04878-6 (PMC12289781; doi:10.1007/s12035-025-04878-6)
Supplement: Supplementary file 2 — Supplementary file2 (PDF 75 KB) [file 12035_2025_4878_MOESM2_ESM.pdf]

Online Resources

Whole transcriptome RNA-Seq reveals drivers of pathological dysfunction in a transgenic model of Alzheimer's disease.

Molecular Neurobiology

Nikita Potemkin[1,2], Sophie M.F. Cawood[1,2], Diane Guévremont[1,2], Bruce Mockett[2,3], Jackson Treece[1], Jo-Ann L. Stanton[1], Joanna M. Williams[1,2*].

[1] Department of Anatomy, School of Biomedical Sciences, University of Otago, P.O. Box 56, Dunedin, New Zealand.

[2] Brain Health Research Centre, Brain Research New Zealand – Rangahau Roro Aotearoa, University of Otago, Dunedin, New Zealand.

[3] Department of Psychology, University of Otago, P.O. Box 56, Dunedin, New Zealand.

[*]Corresponding author: Joanna.williams@otago.ac.nz

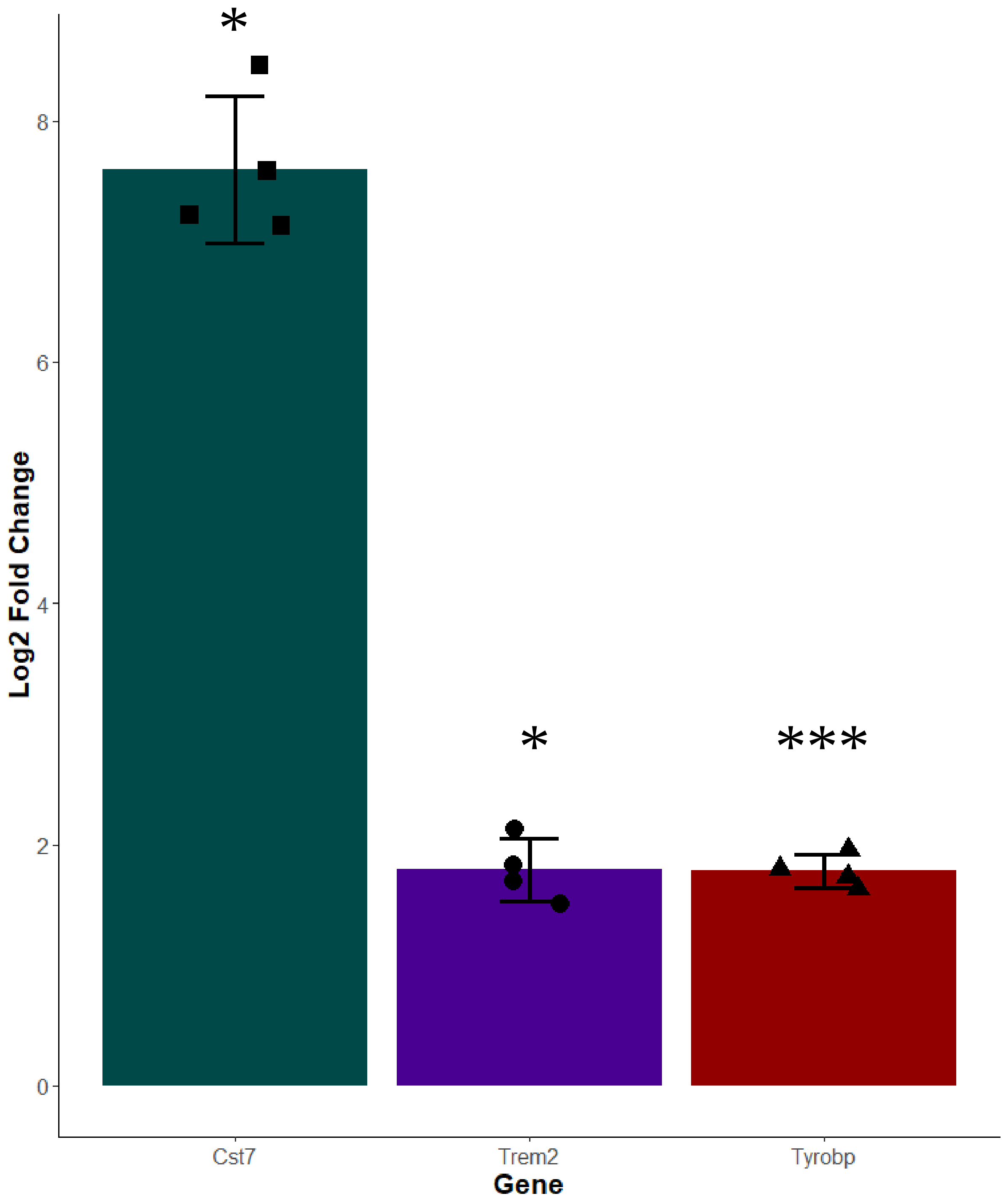

Online Resource 2: Bar and dot plot showing log2 fold-change of the genes Trem2, Tyrobp, and Cst7 in APP/PS1 mice compared to wild-type controls as measured by qPCR. Bars indicate SD, and individual datapoints are also shown. Cst7 reported a 200 ± 97-fold change (log FC 7.7, $p = 0.0016$), while Trem2 and Tyrobp showed fold-changes of 3.5 ± 0.63 and 3.44 ± 0.32 respectively ($p = 0.0059$ and $p < 0.0001$). * = $p<0.01$, *** = $p<0.0001$.
